# Editorial: Development of point-of-care sensors for diagnosis of bacterial-associated infections

**DOI:** 10.3389/fbioe.2025.1716394

**Published:** 2025-12-15

**Authors:** Maria Leilani Torres-Mapa, Janina Bahnemann, Katharina Nikutta, Sofia Arshavsky-Graham

**Affiliations:** 1 Institute of Quantum Optics, Leibniz University Hannover, Hannover, Germany; 2 Lower Saxony Center for Biomedical Engineering, Implant Research and Development (NIFE), Hannover, Germany; 3 Institute of Physics, University of Augsburg, Augsburg, Germany; 4 Department of Prosthetic Dentistry and Biomedical Materials Science, Hannover Medical School, Hannover, Germany; 5 School of Biomedical Engineering, University of British Columbia, Vancouver, BC, Canada

**Keywords:** molecular diagnostics, gas chromatagraphy, point-of-care diagnostics, bacterial infection, artificial intelligence, aptasensor, 3D printed flow chamber, 3D *in vitro *models

## Introduction

Bacterial-associated infections continue to pose significant health burdens globally, exacerbated by rising antimicrobial resistance (AMR) and persistent biofilm-associated complications. Traditional diagnostic methods—culture, polymerase chain reaction (PCR), and enzyme-linked immunoabsorbent assay—are reliable yet often delayed, requiring centralized facilities and specialized personnel. In many contexts, particularly in low-resource settings or during outbreaks, such constraints limit timely patient care. This gap has driven the field toward innovative point-of-care (PoC) diagnostics—compact, affordable, sensitive, and user-friendly tools that align with the WHO’s ASSURED criteria (Affordable, Sensitive, Specific, User-friendly, Rapid/Robust, Equipment-free or simple, Deliverable).

The Research Topic *Development of point-of-care sensors for diagnosis of bacterial-associated infections*, coordinated by Topic Editors Maria Leilani Torres-Mapa (Leibniz University Hannover, Germany), Janina Bahnemann (University of Augsburg, Germany), Katharina Nikutta (Hannover Medical School, Germany), and Sofia Arshavsky Graham (University of British Columbia, Canada) published in Frontiers in Bioengineering and Biotechnology, has brought together a rich Research Topic of eight original research contributions, each advancing PoC biosensing through diverse methodologies and device innovations.

## Highlights from published manuscripts

Among the contributions, one study by Fang et al. reported a loop-mediated isothermal amplification (LAMP) assay targeting the community-acquired respiratory distress syndrome (CARDS) toxin gene of Mycoplasma pneumoniae, achieving nearly perfect concordance with quantitative real-time polymerase chain reaction (qPCR) across 200 clinical respiratory samples. The method is highly specific and showed no cross-reactivity to most common respiratory pathogens or toxins. Its speed, ease of use, and high specificity, positions it well for decentralized diagnostic settings. Another article by Peng et al. applied droplet digital PCR (ddPCR) to enhance early diagnosis, prognosis, and pathogen verification in bloodstream infections among elderly patients, emphasizing its potential in critical care contexts. Using blood culture, as the benchmark for bacterial and fungal bloodstream infections, ddPCR demonstrated high sensitivity and detected most clinically relevant bacterial species. Detection of Helicobacter pylori—a major gastrointestinal pathogen—was addressed via an electrochemical aptasensor by You et al. using silver nanoparticles on graphene oxide with electrodeposited gold nanoparticles, enabling sensitive measurements in blood and stool. The aptasensor exhibited high sensitivity with detection limit ∼3 CFU/mL, high reproducibility as well as high specificity. A further study performed by Kobelt et al. harnessed pyrolysis, gas chromatography and ion mobility spectrometry to rapidly identify several bacteria species present in an oral peri-implant environment, through volatile fragment-derived signatures. The characteristic inverse reduced ion mobility as a function of gas chromatography retention time showed high degree of similarities among the pyrolyzed bacteria but also differences unique to certain bacterial strains. By building a model trained on the measurements from reference bacterial samples, a classification accuracy of over 97% was achieved at the genus level, offering a novel, gas-phase diagnostic route.

Complementing these analytical advances, a systematic review by Brümmer et al. evaluated current three-dimensional (3D) *in vitro* models for implant-associated infections. The review highlights their significance and limitations in understanding the complexity of host-implant-microbe interactions and developing new therapies. Furthermore, the review underscores the importance of creating biomimetic environments, especially relevant for realistic sensor testing and validating PoC platforms. In the realm of non-invasive diagnostics, Jundaeng et al. leveraged artificial intelligence (AI) using a YOLOv8 deep learning model to analyze panoramic radiographs for periodontal disease, demonstrating performance that surpassed trained clinicians in both accuracy and sensitivity. From an engineering perspective, Debener et al. developed a custom 3D-printed, optically accessible flow chamber equipped with integrated sensors which enabled real-time tracking of multispecies oral biofilm growth, advancing capabilities for dynamic biofilm monitoring. Finally, a detection method reported by Tan et al. based on recombinase polymerase amplification (RPA) assay combined with CRISPR/Cas12a successfully identified both *Klebsiella pneumoniae* and its KPC resistance gene. Detection read-outs, among them fluorescence, blue light irradiation, ultraviolet and lateral flow test strips were tested with the latter three methods enabling detection without sophisticated instrument. This work demonstrated the potential of such an assay to be implemented in a highly specific, rapid PoC platform especially relevant for AMR scenario.

Taken together, these eight manuscripts demonstrate the richness of approaches being advanced—from molecular amplification assays and electrochemical platforms to AI-based imaging, 3D models, and CRISPR-powered detection. Each contribution addresses specific gaps in PoC diagnostics while collectively advancing the field toward clinical translation.

## Future perspectives and concluding remarks

The body of work presented in this Research Topic underscores how far PoC sensor development for bacterial infections has progressed in recent years. Emerging themes include the increasing adoption of isothermal amplification technologies, the growing role of artificial intelligence in enhancing diagnostic precision, and the use of 3D models to create more realistic environments for sensor testing. Moreover, platforms integrating CRISPR-based specificity or advanced nanomaterials highlight how molecular biology and materials science can converge to deliver powerful new diagnostic capabilities.

Looking ahead, several priorities are evident. Integration of AI into PoC devices will be key for enabling automated result interpretation and supporting clinical decision-making in real time. The development of multiplexed assays capable of detecting pathogens alongside antimicrobial resistance determinants will be crucial for guiding appropriate therapy. Diagnostic approaches, such as imaging or volatile organic compounds-based sensing, have the potential to greatly improve patient experience and broaden access. Finally, rigorous clinical validation and attention to implementation in low-resource settings will determine the real-world impact of these technologies.

In conclusion, this Research Topic illustrates the extent of innovation currently reshaping the landscape of PoC diagnostics for bacterial-associated infections. By uniting molecular, engineering, computational, and clinical perspectives, these contributions point toward a future in which fast, accurate, and accessible diagnostics will play a central role in combating bacterial infections and mitigating the threat of antimicrobial resistance.

